# Influência do Polimorfismo de Inserção/Deleção do Gene da Enzima Conversora de Angiotensina na Adiposidade e na Função Cardíaca em Pacientes com Insuficiência Cardíaca

**DOI:** 10.36660/abc.20240204

**Published:** 2024-12-16

**Authors:** Marla Darlene Machado Vale, Édina Caroline Ternus Ribeiro, Ingrid da Silveira Knobloch, Ida Vanessa Doederlein Schwartz, Fernanda Sperb-Ludwig, Gabriela Corrêa Souza

**Affiliations:** 1 Programa de Pós-Graduação em Alimentação, Nutrição e Saúde Universidade Federal do Rio Grande do Sul Porto Alegre RS Brasil Programa de Pós-Graduação em Alimentação, Nutrição e Saúde – Universidade Federal do Rio Grande do Sul, Porto Alegre, RS – Brasil; 2 Hospital de Clínicas de Porto Alegre Porto Alegre RS Brasil Hospital de Clínicas de Porto Alegre, Porto Alegre, RS – Brasil; 3 Programa de Pós-Graduação em Cardiologia e Ciências Cardiovasculares Universidade Federal do Rio Grande do Sul Porto Alegre RS Brasil Programa de Pós-Graduação em Cardiologia e Ciências Cardiovasculares – Universidade Federal do Rio Grande do Sul, Porto Alegre, RS – Brasil; 4 Programa de Pós-Graduação em Ciências da Saúde Universidade Federal de Ciências da Saúde de Porto Alegre Porto Alegre RS Brasil Programa de Pós-Graduação em Ciências da Saúde – Universidade Federal de Ciências da Saúde de Porto Alegre, Porto Alegre, RS – Brasil; 5 Programa de Pós-Graduação em Genética e Biologia Molecular Universidade Federal do Rio Grande do Sul Porto Alegre RS Brasil Programa de Pós-Graduação em Genética e Biologia Molecular – Universidade Federal do Rio Grande do Sul, Porto Alegre, RS – Brasil; 6 Departamento de Genética Universidade Federal do Rio Grande do Sul Porto Alegre RS Brasil Departamento de Genética – Universidade Federal do Rio Grande do Sul, Porto Alegre, RS – Brasil

**Keywords:** Polimorfismo Genético, Insuficiência Cardíaca, Adiposidade

## Abstract

**Fundamento:**

O polimorfismo de Inserção/Deleção (I/D) da enzima conversora de angiotensina (ECA) (rs4340) está associado à patogênese da insuficiência cardíaca (IC). Esse polimorfismo pode contribuir para uma maior propensão à IC grave e ao excesso de peso.

**Objetivo:**

Avaliar a adiposidade, a função cardíaca e sua associação com o polimorfismo I/D da ECA em pacientes com IC.

**Métodos:**

Estudo transversal com indivíduos ambulatoriais com idade ≥18 anos diagnosticados com IC. A análise genética foi realizada por reação em cadeia da polimerase seguida de eletroforese em gel de agarose. A fração de ejeção do ventrículo esquerdo (FEVE) foi determinada por ecocardiografia. O estado nutricional foi avaliado pelo índice de massa corporal, enquanto a adiposidade foi avaliada pela análise de Bioimpedância elétrica (BIA), circunferência da cintura, razão cintura-quadril e razão cintura-estatura. O nível de significância adotado foi de 5% (p < 0,05).

**Resultados:**

Setenta e um indivíduos foram incluídos, com média de idade de 55,8 ± 13,0 anos, predominantemente do sexo masculino (66,2%), com classe funcional I e II (90,9%) e FEVE mediana de 30% (24-40). A prevalência de sobrepeso foi de 38%, obesidade classe I foi de 23,9% e obesidade classe II e III foi de 12,7%, com 50,7% apresentando excesso de adiposidade conforme avaliado pela BIA. Um total de 88 alelos D e 54 alelos I do gene da ECA foram identificados. Em relação aos genótipos da ECA, 38,1% eram DD, 47,8% eram ID e 14,1% eram II. Na análise multivariada, o alelo D (genótipos DD+ID versus II) foi associado à FEVE (RP de 0,995; IC de 95% 0,991–1,000; p = 0,048) e à etiologia da IC (cardiomiopatia dilatada: RP 1,283; IC de 95% 1,039–1,583; p = 0,021). Nenhuma associação independente foi encontrada com a adiposidade.

**Conclusão:**

A presença do alelo D do polimorfismo da ECA está associada à FEVE e à etiologia da IC. Apesar da prevalência do sobrepeso na amostra, não foram encontradas associações independentes.

## Graphical abstract

**Figura Central:**
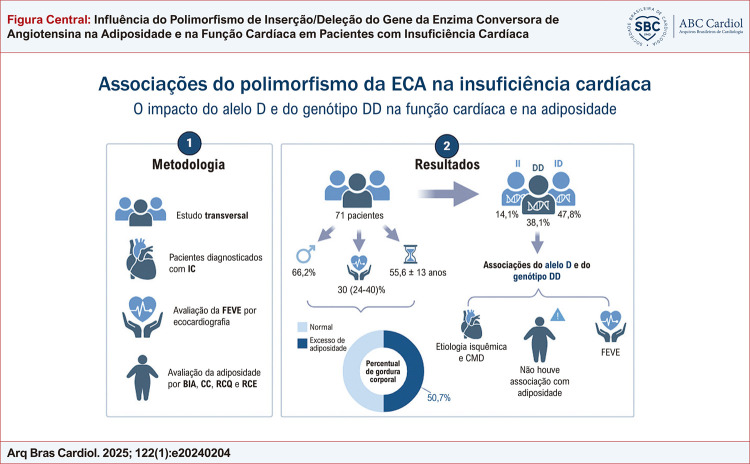
: Influência do Polimorfismo de Inserção/Deleção do Gene da Enzima Conversora de Angiotensina na Adiposidade e na Função Cardíaca em Pacientes com Insuficiência Cardíaca

Associações do polimorfismo da ECA na insuficiência cardíaca. Alelo D: deleção. ECA: enzima conversora de angiotensina; IC: insuficiência cardíaca; FEVE: fração de ejeção do ventrículo esquerdo; BIA: análise de bioimpedância elétrica; CC: circunferência da cintura; RCQ: razão cintura-quadril; RCE: razão cintura-estatura.

## Introdução

A insuficiência cardíaca (IC) é uma síndrome clínica complexa com origens multifatoriais envolvendo fatores genéticos e ambientais.^1 , 2^ Entre os fatores envolvidos na patogênese e progressão da doença está o polimorfismo genético da enzima conversora de angiotensina (ECA).^3 - 5^

A prevalência crescente de IC, juntamente com o rápido aumento da obesidade, representa uma ameaça substancial à saúde.^6^ O acúmulo de tecido adiposo visceral e pericárdico eleva o débito cardíaco e a carga de trabalho. Estima-se que 29% a 40% dos indivíduos com IC estejam acima do peso e 30% a 49% sejam obesos.^2^ Há uma relação dose-dependente entre o índice de massa corporal (IMC) elevado e o risco de IC, sendo a obesidade uma das principais causas de ICFEp.^6^

A Enzima Conversora de Angiotensina-I (ECA 3.4.15.1) regula a pressão arterial (PA) e a homeostase cardiovascular por meio do sistema renina-angiotensina-aldosterona (SRAA).^1 - 3^ O polimorfismo pode estar associado à incidência e progressão da IC, elevando os níveis circulantes de ECA e resultando em aumento da PA, disfunção sistólica, fibrose e remodelação cardíaca.^3 - 5^

O polimorfismo de inserção/deleção (I/D) da ECA (rs4340), localizado no gene da ECA (OMIM 106180) no cromossomo 17, influencia a inserção (alelo I) ou deleção (alelo D) de uma sequência alu de 287 pares de bases no íntron 16.^3^ Esse polimorfismo apresenta três genótipos (II, DD e ID), com o alelo I associado à atividade enzimática reduzida e o alelo D à atividade aumentada, correlacionando-se com a exacerbação da IC e o risco de sobrepeso.^3 - 5^ Além de influenciar a PA, a ECA inibe a diferenciação dos adipócitos, limitando a adipogênese e o armazenamento do tecido adiposo, e resultando em deposição lipídica ectópica, com consequente comprometimento da função cardíaca e contribuição para a disfunção.^4 , 5 - 9^

Embora estudos anteriores associem o polimorfismo da ECA à predisposição à hipertensão arterial sistêmica (HAS),^10^ existe uma escassez de literatura sobre sua associação com a IC. Este estudo visa preencher essa lacuna, explorando a interação entre polimorfismo, função cardíaca e adiposidade em pacientes com IC para fornecer mais informações sobre o manejo da doença, compreendendo a influência do alelo D e do genótipo DD sobre a predisposição ao sobrepeso e exacerbação da função cardiovascular. O objetivo deste estudo é avaliar a adiposidade, a função cardíaca e sua associação com o polimorfismo da ECA em pacientes com IC.

## Métodos

### População do estudo

Trata-se de um desenho transversal, envolvendo indivíduos com idade ≥ 18 anos de ambos os sexos, com diagnóstico estabelecido de IC, recrutados no período entre novembro de 2022 a outubro de 2023 em um ambulatório especializado de um hospital terciário no sul do Brasil. O estudo foi composto por pacientes com IC crônica das classes funcionais I–IV da NYHA ( *New York Heart Association* ).

Critérios de exclusão: a) histórico prévio de infarto agudo do miocárdio; b) insuficiência hepática e renal, em terapia de substituição renal; c) diabetes tipo 1; e d) incapacidade de realizar análise de bioimpedância elétrica (BIA), como no caso de pacientes amputados ou indivíduos com IMC > 39,9 kg/m^2^.

Dados sociodemográficos, comorbidades, tratamento farmacológico, classe funcional da NYHA ( *New York Heart Association* ) e etiologia da IC foram extraídos do prontuário eletrônico e confirmados durante a consulta de pesquisa.

Este estudo foi aprovado pelo Comitê de Ética em Pesquisa do Hospital de Clínicas de Porto Alegre (CAEE: 63243822.2.0000.5327), em conformidade com os princípios da Declaração de Helsinque. Todos os pacientes deram consentimento por escrito.

### Medição da adiposidade e função cardíaca

A avaliação antropométrica incluiu o uso de uma balança digital (Toledo®, Araçatuba, São Paulo, Brasil) para peso corporal e um estadiômetro vertical (Veeder-Root^®^ 2,0 m, São Bernardo do Campo, São Paulo, Brasil) para medição da a estatura.^11^ O IMC foi categorizado de acordo com pontos de corte estabelecidos.^12^

A adiposidade foi avaliada por BIA (tetrapolar, modelo 450, 800 mA, 50 kHz, Biodynamics Corporation, Seattle, Washington, EUA) seguindo o protocolo padrão.^13^ O ponto de corte para análise do excesso de adiposidade foi estabelecido em 38% para mulheres e 27% para homens em relação à massa gorda.^14^

A circunferência cintura (CC) e a circunferência do quadril foram medidas com uma fita métrica inelástica. O risco cardiovascular e o excesso de peso foram determinados com base em valores de referência, incluindo CC > 94 cm para homens e 80 cm para mulheres, razão cintura-quadril (RCQ) ≥ 1,0 para homens e > 0,85 para mulheres e razão cintura-estatura (RCE) > 0,5 para homens e mulheres.^15 - 18^

Resultados de ecocardiografia bidimensional usando o método de Simpson foram empregados para determinar a fração de ejeção do ventrículo esquerdo (FEVE). Essa medida categoriza a IC em três grupos: fração de ejeção reduzida (ICFEr) quando a FEVE é < 40%, fração de ejeção intermediária (ICFEi) com FEVE entre 40-49% e fração de ejeção preservada (ICFEp), com FEVE é ≥ 50%.^1 , 2^

### Análise genética

Amostras de sangue (4 mL) com EDTA como anticoagulante foram coletadas e armazenadas. A extração de DNA foi realizada usando o kit Easy DNA Purification™ (Invitrogen™), seguido pela amplificação do polimorfismo ECA por reação em cadeia da polimerase (PCR). Os produtos de PCR foram genotipados por eletroforese em gel de agarose. Os primers usados foram: 5’-CTG GAG AGC CAC TCC CAT CCT TTC T-3’ e 5’-GAC GTG GCC ATC ACA TTC GTC AGA T-3’. As condições de PCR foram as seguintes: desnaturação inicial a 95 °C por 5 minutos; seguido por ciclos a 95°C por 40 segundos, 62 °C por 45 segundos, 72 °C por 45 segundos e uma extensão final a 72 °C por 5 minutos. Fragmentos de polimorfismo da ECA com deleção (alelo D) e sem deleção (alelo I) de 190 e 490 pares de bases, respectivamente, foram visualizados em um gel de agarose a 1,5% contendo coloração de ácido nucleico GelRed (Sigma-Aldrich).

### Análise estatística

As variáveis contínuas foram descritas como média ± desvio padrão ou mediana e intervalo interquartil (percentis 25 a 75) de acordo com a normalidade dos dados, que foi avaliada usando o teste de Shapiro-Wilk. O teste t de Student não pareado ou ANOVA unidirecional com Tukey post-hoc para amostras independentes foi aplicado para a comparação de médias. Em caso de assimetria, o teste de Mann-Whitney ou Kruskal-Wallis foi usado. No entanto, no teste de Kruskal-Wallis, o teste post hoc de Dunn não foi conduzido porque não houve valor-p < 0,05. Variáveis categóricas foram descritas por frequência absoluta/relativa e o teste qui-quadrado de Pearson ou teste exato de Fisher foi usado para avaliar associações. Em caso de significância, a análise residual identificou associações. Frequências genéticas foram testadas para equilíbrio de Hardy-Weinberg.

Os grupos foram formados de acordo com os três genótipos (II, DD e ID) do polimorfismo da ECA. Para analisar o efeito independente do alelo D, os participantes foram divididos em dois grupos: aqueles com a presença do alelo D (genótipos DD + ID) e aqueles sem o alelo D (somente genótipo II).

A interação dos alelos da ECA e variáveis de interesse foi analisada por regressão de Poisson, com um estimador robusto usado para controlar fatores de confusão. Na análise multivariada, variáveis de interesse com um valor-p < 0,20 na análise univariada foram consideradas.

O nível de significância adotado foi de 5% (p < 0,05) e as análises foram realizadas usando o SPSS 21.0 (IBM Corp., Armonk, NY, EUA).

## Resultados

Foram incluídos 71 pacientes, sendo 66,2% do sexo masculino, com média de idade de 55,8 ± 13 anos. A maioria dos pacientes apresentou classes funcionais NYHA I e II (90,9%) e FEVE mediana de 30% (24-40). Em relação à prevalência de sobrepeso e obesidade, 38% dos pacientes estavam acima do peso, 23,9% tinham obesidade classe I e 12,7% tinham obesidade classe II e III. A BIA revelou que 50,7% dos pacientes apresentavam excesso de adiposidade. Parâmetros antropométricos incluindo CC, RCQ e RCE indicaram um risco cardiovascular aumentado e excesso de peso, conforme mostrado na Tabela 1 .

**Tabela 1:** – Características clínicas, demográficas e antropométricas dos genótipos do polimorfismo da ECA e presença ou ausência do alelo D em indivíduos com insuficiência cardíaca

		Genótipo				Alelo D		
	Total	DD	ID	II	p	Presença	Ausência	p
**N.º de pacientes**	71	27	34	10		61	10	
**Sexo (%)**								
Feminino	24 (33,8)	6 (22,2)	16 (47,1)	2 (20,0)	0,077^§^	22 (36,1)	2 (20,0)	0,477^§^
Masculino	47 (66,2)	21 (77,8)	18 (52,9)	8 (80,0)		39 (63,9)	8 (80,0)	
**Idade (anos)**	55,8 ± 13,0	57,2 ± 12,3	56,1 ± 12,4	50,8 ± 16,7	0,404*	56,6 ± 12,3	50,8 ± 16,7	0,191^†^
**Ano de escolaridade**	8 (4,0 – 11,0)	8 (4,0 – 11,0)	8 (4,0 – 11,0)	8 (4,0 – 9,0)	0,940^‡^	8 (4,0 – 11,0)	8 (4,0 – 9,0)	0,752^//^
**Etnia (%)**								
Branco	64 (90,1)	24 (88,9)	31 (91,2)	9 (90,0)	1,000^§^	55 (90,2)	9 (90,0)	1,000^§^
Não branco	7 (9,9)	3 (11,1)	3 (8,8)	1 (10,0)		6 (9,8)	1 (10,0)	
**Número de comorbidades**	2 (1,0 – 3,0)	1 (1,0 – 3,0)	2 (1,0 – 2,0)	0,5 (0,0 – 3,0)	0,321^‡^	2 (1,0 – 2,5)	0,5 (0,0 – 3,0)	0,198^//^
**Comorbidades (%)**								
DM	20 (28,2)	9 (33,3)	8 (23,5)	3 (30,0)	0,693^§^	17 (27,9)	3 (30,0)	1,000^§^
HAS	26 (36,6)	8 (29,6)	14 (41,2)	4 (40,0)	0,631^§^	22 (36,1)	4 (40,0)	1,000^§^
DRC	7 (9,9)	3 (11,1)	3 (8,8)	1 (10,0)	1,000^§^	6 (9,8)	1 (10,0)	1,000^§^
DLP	5 (7,0)	3 (11,1)	2 (5,9)	0 (0,0)	0,688^§^	5 (8,2)	0 (0,0)	1,000^§^
**Tempo desde o diagnóstico de IC (%)**								
≤ 18 meses	16 (24,6)	5 (19,2)	6 (20,0)	5 (55,6)	0,067^§^	11 (19,6)^a^	5 (55,6)b	**0,034** ^**§**^
≥ 18 meses	49 (75,4)	21 (80,8)	24 (80,0)	4 (44,4)		45 (80,4)^b^	4 (44,4)a	
**Etiologia da IC (%)**								
Isquêmica	7 (11,1)	5 (20,80)a	1 (3,40)a	1 (10,0)a	**0,009** ^**§**^	6 (11,3)^a^	1 (10,0)^a^	**0,025** ^**§**^
Cardiomiopatia Dilatada	12 (19,0)	1 (4,20)^a^	6 (20,70)^b^	5 (50,0)^a.b^		7 (13,2)^a^	5 (50,0)^b^	
Outras etiologias	44 (69,8)	18 (75,50)a	22 (75,90)a	4 (40,0)a		40 (75,5)^a^	4 (40,0)^b^	
**FEVE (%)**	30 (24,0 – 40,0)	32 (25,0 – 45,0)	30 (23,0 – 40,0)	24 (18,5 – 33,2)	0,114^‡^	31 (24,5 – 41,0)	24 (18,5 – 33,2)	**0,050** ^**//**^
**Classificação da FEVE (%)**								
ICFEr	51 (71,8)	17 (63,0)	25 (73,5)	9 (90,0)	0,682^§^	42 (68,9)	9 (90,0)	0,564^§^
ICFEi	12 (16,9)	6 (22,2)	5 (14,7)	1 (10,0)		11 (18,0)	1 (10,0)	
ICFEp	8 (11,3)	4 (14,8)	4 (11,8)	0 (0,0)		8 (13,1)	0 (0,0)	
**Classificação NYHA (%)**								
I e II	60 (90,9)	25 (96,2)	30 (90,9)	5 (71,4)	0,137^§^	55 (93,2)	5 (71,4)	0,118^§^
III e IV	6 (9,1)	1 (3,8)	3 (9,1)	2 (28,6)		4 (6,80)	2 (28,6)	
**Número de medicamentos**	7 (6,0 – 9,0)	8 (6,0 – 11,0)	7 (5,7 – 9,2)	6,5 (5,7 – 8,0)	0,365^‡^	7 (6,0 –10,0)	7 (4,0 – 8,3)	0,294^//^
**Medicamentos (%)**								
IECA/BRA/INRA	58 (81,7)	24 (88,9)	26 (76,5)	8 (80,0)	0,443^§^	50 (82,0)	8 (80,0)	0,881^§^
Betabloqueadores	69 (97,2)	26 (96,3)	33 (97,1)	10 (100,0)	1,000^§^	59 (96,7)	10 (100,0)	1,000^§^
Diuréticos	58 (81,7)	23 (85,2)	58 (81,7)	7 (70,0)	0,584^§^	51 (83,6)	7 (70,0)	0,376^§^
Espironolactona	56 (78,9)	22 (81,5)	25 (73,5)	9 (90,0)	0,448^§^	48 (77,0)	9 (90,0)	0,448^§^
**TFG (ml/min)**	71,0 ± 26,3	69,8 ± 27,2	68,3 ± 25,8	83,2 ± 25,0	0,286*	69,0 ± 26,2	83,2 ± 25,0	0,115^†^
**Creatinina (mg/dL)**	1,0 (0,8 –1,4)	1,2 (0,9 – 1,4)	1,1 (0,8 – 1,5)	1,0 (0,7 – 1,2)	0,557*	1,2 (0,9 – 1,5)	1,0 (0,7 –1,3)	0,305^‡^
**Peso (kg)**	79,9 ± 17,1	84,1 ± 15,5	76,2 ± 17,0	81,1 ± 19,7	0,189*	79,7 ± 16,8	81,1 ± 19,7	0,812^†^
**IMC (kg/m^2^)**	28,8 ± 4,9	30,0 ± 4,5	27,9 ± 5,3	28,6 ± 4,9	0,274*	28,9 ± 5,0	28,6 ± 4,9	0,878^†^
**Classificação BMI (%)**								
Normal	18 (25,4)	4 (14,8)	12 (35,3)	2 (20,0)	0,169^§^	16 (26,2)	2 (20,0)	0,224^§^
Sobrepeso	27 (38,0)	12 (44,4)	12 (35,3)	3 (30,0)		24 (39,3)	3 (30,0)	
Obesidade Classe I	17 (23,9)	5 (18,5)	7 (20,6)	5 (50,0)		12 (19,7)	5 (50,0)	
Obesidade Classe II e III	9 (12,7)	6 (22,2)	3 (8,8)	0 (0,0)		9 (14,8)	0 (0,0)	
**CC (cm)**	101,4 ± 13,3	102,2 ± 13,3	102,2 ± 13,3	102,2 ± 13,3	0,145*	101,33 ± 13,0	102,4 ± 15,6	0,832^†^
**RCQ (cm)**	0,9 ± 0,1	1,0 ± 0,1	0,9 ± 0,1	0,9 ± 0,1	0,218*	0,9 ± 0,1	0,9 ± 0,1	0,968^†^
**RCE (cm)**	0,6 ± 0,1	0,6 ± 0,1	0,6 ± 0,1	0,6 ± 0,1	0,241*	0,6 ± 0,7	0,6 ± 0,7	0,892^†^
**Gordura Corporal (%)**	29,9 ± 6,3	29,7 ± 5,9	30,7 ± 6,9	28,1 ± 5,2	0,528*	30,2 ± 6,5	28,1 ± 5,2	0,327^†^
Homens	27,7 ± 5,0	27,7 ± 4,9	27,6 ± 5,7	27,6 ± 3,7	0,997^§^	27,7 ± 5,2	27,6 ± 3,7	0,944^§^
Mulheres	34,81 ± 6,2	36,7 ± 3,1	34,8 ± 6,6	30,3 ± 11,6	0,461^§^	35,2 ± 5,7	30,3 ± 11,6	0,298^§^
**Classificação da adiposidade (%)**								
Normal	34 (49,3)	14 (51,9)	15 (46,9)	5 (50,0)	0,929^§^	29 (49,2)	5 (50,0)	1,000^§^
Excesso de adiposidade	35 (50,7)	13 (48,1)	17 (53,1)	5 (50,0)		30 (50,8)	5 (50,0)	
**Gordura Corporal (kg)**	24,5 ± 8,2	25,3 ± 7,8	24,1 ± 8,6	23,2 ± 8,4	0,754*	24,7 ± 8,2	23,2 ± 8,4	0,613^†^

Dados expressos como média ± desvio padrão ou mediana e (percentis 25 e 75); *ANOVA unidirecional; ^†^Teste t de Student; ^‡^Kruskal-Wallis; ^§^Teste qui-quadrado ou teste exato de Fisher; ^//^Mann-Whitney; Presença do alelo D: genótipos DD + ID; Ausência do alelo D: genótipo II; Associação estatisticamente significativa pelo teste de resíduos ajustados em nível de significância de 5%, letras diferentes: diferença estatisticamente significativa entre os grupos p < 0,005; INRA: inibidor da neprilisina/receptor da angiotensina; DM: Diabetes Mellitus; HAS: hipertensão arterial sistêmica; DRC: doença renal crônica; DLP: dislipidemia; IECA: inibidor da enzima conversora de angiotensina; BRA: Bloqueadores dos Receptores da Angiotensina; FEVE: fração de ejeção do ventrículo esquerdo; ICFEr: insuficiência cardíaca com fração de ejeção reduzida; ICFEi: insuficiência cardíaca com fração de ejeção intermediária; ICFEp: insuficiência cardíaca com fração de ejeção preservada; NYHA: New York Heart Association; TFG: Taxa de Filtração Glomerular; CC: circunferência da cintura; RCQ: razão cintura-quadril; RCE: razão cintura-estatura.

### Genótipo e frequências de alelo

A frequência alélica foi de 88 alelos D e 54 alelos I. As distribuições genotípicas foram 38,1% para DD, 47,8% para ID e 14,1% para II. As frequências do polimorfismo I/D da ECA estavam em equilíbrio de Hardy-Weinberg.

### Características clínicas e antropométricas no polimorfismo da ECA

A análise univariada foi conduzida para avaliar potenciais variáveis de confusão ( Tabela 1 do material suplementar). Na análise multivariada, ao avaliar a associação do alelo D com as variáveis de interesse, verificou-se que a FEVE (p = 0,048) e a etiologia da cardiomiopatia dilatada (CMD) (p = 0,021) estavam associadas à presença do alelo D (DD+ID versus II) em comparação aos indivíduos sem o alelo D. Ao avaliar especificamente a influência do genótipo DD entre os demais genótipos, verificou-se que a CMD (p = 0,020) e a etiologia isquêmica (p = 0,024) estavam associadas ao genótipo DD em comparação ao genótipo ID. Além disso, essas associações entre FEVE (p = 0,013), etiologia da CMD (p < 0,001) e o genótipo DD persistem quando comparadas ao genótipo II. Outras variáveis, como estado nutricional, CC, RCQ, RCE e percentual de gordura corporal, não apresentaram associações significativas na análise multivariada, conforme descrito na Tabela 2 .

**Tabela 2:** – Análise de regressão de Poisson multivariada entre genótipos de polimorfismo da ECA e presença ou ausência do alelo D, características clínicas e adiposidade em indivíduos com insuficiência cardíaca

		DD + ID vs II			DD vs ID			DD vs II	
	**RP**	**IC de 95%**	p*	**RP**	**IC de 95%**	**p^†^**	**RP**	**IC de 95%**	**p^‡^**
**FEVE (%)**	0,995	0,991 - 1,000	**0,048**	0,996	0,990 - 1,001	0,118	0,989	0,980 - 0,998	**0,013**
**Classificação da FEVE (%)**									
ICFEr	1,145	1,040 - 1,261	**0,006**	1,193	0,941 0 1,512	0,144	1,411	1,132 - 1,758	**0,002**
ICFEi	1,060	0,925 - 1,216	0,402	1,060	0,782 - 1,436	0,709	1,166	0,926 - 1,468	0,192
ICFEp	1			1			1		
**Etiologia da IC (%)**									
Isquêmica	1,047	0,820 - 1,337	0,714	0,755	0,591 - 0,964	**0,024**	0,969	0,747 - 1,257	0,813
Cardiomiopatia Dilatada	1,283	1,039 - 1,583	**0,021**	1,216	1,032 - 1,433	**0,020**	1,584	1,260 - 1,992	**< 0,001**
Outras etiologias	1			1			1		
**IMC (Kg/m^2^)**	0,999	0,986 - 1,013	0,879	0,988	0,972 - 1,005	0,154	0,968	0,968 - 1,015	0,469
**Classificação BMI (%)**									
Normal	1			1			1		
Sobrepeso	0,985	0,832 - 1,165	0,857	0,873	0,727- 1,047	0,143	0,958	0,686 - 1,338	0,802
Obesidade Classe I	1,169	0,952 - 1,437	0,136	0,893	0,733- 1,087	0,259	1,212	0,868- 1,692	0,260
Obesidade Classe II e III	0,906	0,789 - 1,039	0,157	0,774	0,573 - 1,046	0,096	0,806	0,605 - 1,073	0,140
**CC (cm)**	1,000	0,994 - 1,006	0,969	0,995	0,988 - 1,002	0,132	0,999	0,989 - 1,008	0,763
**RCQ**	0,815	0,269 - 2,473	0,718	0,729	0,216 - 2,456	0,610	0,658	0,137 - 3,168	0,602
**RCE**	0,882	0,333 - 2,335	0,800	0,432	0,146 - 1,282	0,131	0,572	0,121 - 2,711	0,482
**Gordura Corporal (%)**	0,989	0,973 - 1,005	0,185	0,998	0,983 - 1,014	0,837	0,993	0,970 - 1,017	0,563

Razão de prevalência (RP) e intervalo de confiança (IC) de 95%; IC: insuficiência cardíaca; FEVE: fração de ejeção do ventrículo esquerdo; ICFEr: insuficiência cardíaca com fração de ejeção reduzida; ICFEi: insuficiência cardíaca com fração de ejeção intermediário; ICFEp: insuficiência cardíaca com fração de ejeção preservada; IMC: índice de massa corporal; CC: circunferência da cintura; RCQ: razão cintura-quadril; RCE: razão cintura-estatura. Modelo multivariável - FEVE: ajustada para sexo e betabloqueador*, ^‡^ ou IECA/BRA/INRA^†^; Classificação da FEVE, etiologia da IC: ajustada para sexo e betabloqueador*, ^‡^ ou IECA/BRA/INRA^†^; Gordura corporal, CC, RCQ, RCE, IMC, Estado nutricional: ajustado para sexo, betabloqueador*, ^‡^ ou IECA/BRA/INRA†, e classificação da FEVE*, ^‡^ ou etiologia da IC^†^.

## Discussão

Os principais achados deste estudo são a associação entre a presença do alelo D e o genótipo DD do gene da ECA com FEVE e etiologia em pacientes com IC. O excesso de adiposidade foi prevalente na amostra, porém, não foram identificadas associações independentes com polimorfismo.

Em contraste com estudos anteriores, que indicaram redução da FEVE em pacientes com o genótipo DD,^3 , 18 , 19^ nosso estudo apresenta uma perspectiva diferente, indicando que a FEVE mediana é menor em pacientes sem a presença do alelo D, possivelmente relacionada ao menor tempo de diagnóstico. Isso ocorre porque os portadores do alelo D têm um tempo de diagnóstico maior e, como resultado, o bloqueio neuro-humoral (interação farmacogenética entre o uso de betabloqueador e inibidores da ECA)^19^ pode ter neutralizado a atividade excessiva do SRAA secundária à DD, influenciando essa diferença entre os grupos. No entanto, ao analisar a associação entre a presença do alelo D ou do genótipo DD versus II, observou-se que a FEVE aumentou em associação com a diminuição da prevalência do alelo. Sendo assim, a relevância clínica deste achado implica que pacientes com o alelo D e genótipo DD, quando comparados ao II, apresentam uma deterioração mais pronunciada da função cardíaca.

O perfil genotípico da ECA na população estudada mostra baixa prevalência do genótipo II em comparação com estudos internacionais, nos quais uma prevalência de 23% foi identificada.^20^ Ao comparar esses resultados com estudos nacionais, a prevalência do II foi ainda menor, representando apenas 3% a 4,5% da população.^3 , 18^ Dado que essa alteração genômica é relativamente recente, a inserção dessa sequência Alu ainda não está completamente estabelecida no genoma humano, apresentando também variações na distribuição entre diferentes grupos étnicos.^21^ Portanto, essa baixa prevalência do genótipo II pode estar relacionada a essa característica da população.

Nosso estudo revelou predomínio de indivíduos de descendência europeia (90,1%). Em contrapartida, estudos nacionais realizados em outras regiões do país^3 , 18^ mostraram maior percentual de indivíduos de descendência africana (14,1% a 36%) e de outras etnias (12,6% a 16,2%). Isso evidencia a heterogeneidade epidemiológica presente no Brasil; entretanto, a região sul tem uma proporção maior de indivíduos de descendência europeia. Em relação aos outros genótipos, observamos um alinhamento com dados europeus, mostrando uma prevalência maior do genótipo ID.^19^

Outro achado foi a associação da etiologia da CMD com uma prevalência aumentada do alelo D e genótipo DD em pacientes com IC, corroborando achados anteriores^22 , 23^ que estabeleceram essa relação entre o polimorfismo da ECA e a disfunção sistólica do ventrículo esquerdo. Isso pode ser atribuído à ativação neuro-humoral mais intensa do SRAA.^3 , 18 , 19^ O polimorfismo I/D da ECA desempenha um papel significativo, contribuindo com cerca de 50% para a variação nos níveis circulantes de ECA.^22 , 23^ O alelo D intensifica a atividade da ECA, aumentando a síntese de angiotensina e potencializando a ativação do SRAA, o que resulta em dilatação cardíaca.^24^ Além disso, a etiologia isquêmica da IC foi associada a uma redução na prevalência do genótipo DD em comparação ao ID, possivelmente devido à ausência de influência do polimorfismo da ECA em doenças cardíacas isquêmicas.^25^

Embora o genótipo DD tenha sido previamente associado ao risco de sobrepeso/obesidade na população em geral,^8^ devido à sua interação com o crescimento e a função dos adipócitos, a relação entre o polimorfismo e o sobrepeso foi explorada em pacientes hipertensos, apresentando resultados conflitantes.^24 , 26^ No entanto, no presente estudo, nenhuma associação independente de polimorfismo com adiposidade foi observada, o que pode ser devido à alta prevalência de sobrepeso/obesidade na amostra em comparação a outras populações. As diferenças no IMC médio dos genótipos da ECA entre nosso estudo e o de Sun et al.^24^ foram, respectivamente: 30,0 kg/m^2^ vs 26,1 kg/m^2^ para DD; 27,9 kg/m^2^ vs 26,0 kg/m^2^ para ID; e 28,6 kg/m^2^ vs 25,9 kg/m^2^ para II.

A prevalência significativa de excesso de adiposidade entre pacientes com IC é um fator preocupante, dada a considerável ameaça à saúde que a obesidade representa. O acúmulo de tecido adiposo, especialmente na região visceral e gordura pericárdica, resulta em aumento do débito cardíaco e da carga de trabalho.^1 , 2 , 8^ Estudos recentes apontam para uma relação dose-dependente entre o aumento do IMC e o risco de IC, sendo a obesidade considerada uma das principais causas de ICFEp.^6 , 9^ Nesse cenário, é crucial implementar intervenções eficazes, incluindo monitoramento nutricional, visando estimular a redução de peso e mitigar os riscos relacionados à adiposidade.

Possíveis limitações deste estudo incluem o número restrito de participantes, especialmente no grupo de genótipo II, possivelmente relacionado à gravidade da população predominantemente composta por indivíduos com genótipos DD e ID. O método padrão-ouro para análise da composição corporal é a absorciometria de raios X de dupla energia (DEXA). Na sua ausência, a BIA foi usada. No entanto, a BIA apresenta certas limitações, como a dificuldade em distinguir com precisão entre água extracelular e intracelular. Isso pode tornar a estimativa da massa corporal suscetível a desequilíbrios eletrolíticos ou variações no estado de hidratação, que são comuns em pacientes com IC. Em nosso estudo, protocolos específicos foram adotados para minimizar essas limitações e, na presença de edema, o teste não foi realizado. Embora este seja o primeiro estudo a avaliar o polimorfismo da ECA em pacientes brasileiros com IC e vários resultados relevantes tenham sido obtidos com significância estatística, há uma necessidade evidente de estudos futuros com uma amostra mais ampla, capaz de explorar essas e outras variáveis potencialmente relacionadas à gravidade da IC e a alteração na composição corporal.

## Conclusão

Em resumo, este estudo demonstrou que a presença do alelo D do polimorfismo da ECA está associada à etiologia da FEVE e da IC. Apesar da prevalência do sobrepeso na amostra, não foram encontradas associações independentes.

*Material suplementar

Para informação adicional, por favor, clique aqui.


