# Supplementary material for: Influência do Polimorfismo de Inserção/Deleção do Gene da Enzima Conversora de Angiotensina na Adiposidade e na Função Cardíaca em Pacientes com Insuficiência Cardíaca
Source: Arq Bras Cardiol. 2024 Dec 16;122(1):e20240204. [Article in Portuguese] doi: 10.36660/abc.20240204 (PMC11841369; doi:10.36660/abc.20240204)
Supplement: Supplementary file 1 [file 2024-0204_AO_SUPPLEMENTARY_MATERIAL.pdf]

SUPPLEMENTARY MATERIAL

Table 1 - Univariate Poisson regression analysis between ACE polymorphism genotypes and presence or absence of the D allele, clinical characteristics, and adiposity in individuals with heart failure

	DD + ID *vs* II			DD *vs* ID			DD *vs* II		
	**Pr**	**CI 95%**	***P***	**Pr**	**CI 95%**	***P***	**Pr**	**CI 95%**	***P***
**Gender (%)**									
Female	0.926	0.807 - 1.062	0.271	0.926	0.807 - 1.062	0.271	0.926	0.807 - 1.062	0.271
Male	1			1			1		
**Age (years)**	0.996	0.990 - 1.003	0.291	0.999	0.992 - 1.005	0.715	0.995	0.985 - 1.004	0.276
**Ethnicity (%)**									
White	0.998	0.786 – 1.267	0.987	1.042	0.788 – 1.379	0.771	1.018	0.710 – 1.459	0.922
Non-White	1			1			1		
**Etiology of HF (%)**									
Ischemic	1.048	0.824 – 1.332	0.704	0.753	0.572 – 0.990	**0.042**	0.987	0.739 – 1.319	0.930
Dilated Cardiomyopathy	1.299	1.051 - 1.605	**0.016**	1.198	1.009– 1.422	**0.039**	1.551	1.255 – 1.919	**<0.001**
Other etiologies	1			1			1		
**LVEF (%)**	0.995	0.991 – 0.999	**0.015**	0.998	0.992– 1.004	0.551	0.991	0.984 – 0.997	**0.003**
**LVEF Classification (%)**									
HFrEF	1.176	1.076 – 1.286	**<0.001**	1.063	0.829 – 1.364	0.628	1.346	1.175 – 1.542	**<0.001**
HFmrEF	1.083	0.938 – 1.252	0.277	0.970	0.713 – 1.318	0.844	1.143	0.911 – 1.434	0.249
HFpEF	1			1			1		

**NYHA Classification (%)**									
I and II	0.812	0.608 -1.086	0.161	0.883	0.683-1.142	0.343	0.883	0.683-1.142	0.343
III and IV	1			1			1		
**Medications (%)**									
ACEI/ARB/ARNI									
Yes	1			1			1		
No	1.014	0.841 - 1.223	0.884	0.897	0.737 - 1.051	**0.158**	0.893	0.642 - 1.241	0.500
Beta-Blocker									
Yes	1			1			1		
No	0.873	0.812 – 0.939	**<0.001**	0.962	0.602– 1.538	0.871	0.783	0.698 – 0.878	**<0.001**
Diuetics									
Yes	1			1			1		
No	1.098	0.899 - 1.342	0.360	1.033	0.838 - 1.273	0.762	1.158	0.872 - 1.539	0.311
Spironolactone									
Yes	1			1			1		
No	0.919	0.795 – 1.062	0.252	1.072	0.897– 1.283	0.444	0.904	0.681– 1.201	0.487
**BMI (kg/m^2)**	0.999	0.985 – 1.012	0.868	0.987	0.973 – 1.001	0.078	0.989	0.967 – 1.012	0.362
**BMI Classification (%)**									
Normal	1			1			1		
Overweight	1.000	0.845 - 1.184	1.000	0.857	0.716 - 1.026	0.097	0.900	0.647 - 1.251	0.531
Obesity Class I	1.165	0.942 - 1440	0.159	0.905	0.731 – 1.121	0.359	1.125	0.793 – 1.597	0.510
Obesity Class II and III	0.900	0.790 - 1.026	0.114	0.762	0.587- 0.989	**0.041**	0.750	0.565- 0.995	**0.046**

**WC (cm)**	1.001	0.995 - 1.007	0.844	0.994	0.984 – 0.999	**0.012**	0.997	0.987 – 1.007	0.582
**WHR**	1.018	0.376 – 2.758	0.972	0.391	0.170 – 0.897	**0.027**	0.613	0.136 – 2.758	0.524
**WHtR**	0.931	0.344 – 2.521	0.888	0.372	0.136– 1.016	0.054	0.460	0.085 – 2.483	0.367
**Body Fat (%)**	0.994	0.984 – 1.004	0.252	1.004	0.991– 1.016	0.566	0.992	0.974– 1.011	0.412
**Body Fat (kg)**	0.998	0.989 – 1.007	0.605	0.997	0.9887– 1.007	0.568	0.995	0.980– 1.009	0.476

PR (Prevalence Ratio) and 95% CI (Confidence Interval). ARNI=Angiotensin receptor–neprilysin inhibitor. LVEF = Left ventricular ejection fraction. HFrEF = Heart failure with reduced ejection fraction. HFmrEF = Heart failure with mid-range ejection fraction. HFpEF = Heart failure with preserved ejection fraction. NYHA = New York Heart Association. ACEI = Angiotensin-converting enzyme inhibitor. ARB = Angiotensin receptor blocker. BMI = Body mass index. WC = Waist circumference. WHR = Waist-to-hip ratio. WHtR = Waist-to-height ratio.
