# Supplementary material for: Influência do Polimorfismo de Inserção/Deleção do Gene da Enzima Conversora de Angiotensina na Adiposidade e na Função Cardíaca em Pacientes com Insuficiência Cardíaca
Source: Arq Bras Cardiol. 2024 Dec 16;122(1):e20240204. [Article in Portuguese] doi: 10.36660/abc.20240204 (PMC11841369; doi:10.36660/abc.20240204)
Supplement: Translation

# Translation: Influence of Insertion/Deletion Polymorphism of the Angiotensin Converting Enzyme Gene on Adiposity and Cardiac Function in Patients with Heart Failure

**DOI:** 10.36660/abc.20240204i

**Authors:** Marla Darlene Machado Vale, Édina Caroline Ternus Ribeiro, Ingrid da Silveira Knobloch, Ida Vanessa Doederlein Schwartz, Fernanda Sperb-Ludwig, Gabriela Corrêa Souza

**Affiliations:** 1 Brazil Programa de Pós-Graduação em Alimentação, Nutrição e Saúde – Universidade Federal do Rio Grande do Sul, Porto Alegre, RS – Brazil; 2 Brazil Hospital de Clínicas de Porto Alegre, Porto Alegre, RS – Brazil; 3 Brazil Programa de Pós-Graduação em Cardiologia e Ciências Cardiovasculares – Universidade Federal do Rio Grande do Sul, Porto Alegre, RS – Brazil; 4 Brazil Programa de Pós-Graduação em Ciências da Saúde – Universidade Federal de Ciências da Saúde de Porto Alegre, Porto Alegre, RS – Brazil; 5 Brazil Programa de Pós-Graduação em Genética e Biologia Molecular – Universidade Federal do Rio Grande do Sul, Porto Alegre, RS – Brazil; 6 Brazil Departamento de Genética – Universidade Federal do Rio Grande do Sul, Porto Alegre, RS – Brazil

**Keywords:** Genetic Polymorphism, Heart Failure, Adiposity

## Abstract

**Background:**

The angiotensin-converting enzyme (ACE) insertion/deletion (I/D) polymorphism (rs4340) is associated with the pathogenesis of heart failure (HF). This polymorphism may contribute to a greater propensity for severe HF and excess weight.

**Objective:**

To evaluate adiposity, cardiac function, and their association with ACE I/D polymorphism in HF patients.

**Methods:**

Cross-sectional study with ambulatory individuals ≥18 years diagnosed with HF. Genetic analysis was performed using polymerase chain reaction followed by agarose gel electrophoresis. Left ventricular ejection fraction (LVEF) was determined by echocardiography. Nutritional status was assessed using body mass index, while adiposity was analyzed using bioelectrical impedance analysis (BIA), waist circumference, waist-to-hip ratio, and waist-to-height ratio. The adopted significance level was 5% (p < 0.05).

**Results:**

Seventy-one individuals were included, with a mean age of 55.8 ± 13.0 years, predominantly male (66.2%), with functional class I and II (90.9%), and a median LVEF of 30% (24-40). The prevalence of overweight was 38%, class I obesity was 23.9%, and class II and III obesity was 12.7%, with 50.7% exhibiting excess adiposity as assessed by BIA. A total of 88 D alleles and 54 I alleles of the ACE gene were identified. Regarding ACE genotypes, 38.1% were DD, 47.8% were ID, and 14.1% were II. In the multivariate analysis, the D allele (DD + ID genotypes versus II) was associated with LVEF (PR 0.995; 95% CI 0.991–1.000; p = 0.048) and with the etiology of HF (dilated cardiomyopathy: PR 1.283; 95% CI 1.039–1.583; p = 0.021). No independent association was found with adiposity.

**Conclusion:**

The presence of the D allele of the ACE polymorphism is associated with LVEF and HF etiology. Despite overweight being prevalent in the sample, no independent associations were found.

## Introduction

Heart failure (HF) is a complex clinical syndrome with multifactorial origins involving genetic and environmental factors.^1 , 2^Among the factors involved in the pathogenesis and progression of the disease is the genetic polymorphism of the angiotensin-converting enzyme (ACE).^3 - 5^

The increasing prevalence of HF, along with the rapid rise in obesity, poses a substantial health threat.^6^The accumulation of visceral and pericardial adipose tissue elevates cardiac output and workload. It is estimated that 29% to 40% of individuals with HF are overweight, and 30% to 49% are obese.^2^There is a dose-dependent relationship between elevated BMI and the risk of HF, with obesity being one of the main causes of HFpEF.^6^

Angiotensin-converting Enzyme-I (ACE 3.4.15.1) regulates Blood Pressure (BP) and cardiovascular homeostasis through the renin-angiotensin-aldosterone system (RAAS).^1 - 3^The polymorphism can be associated with the incidence and progression of HF, elevating circulating levels of ACE and resulting in increased BP, systolic dysfunction, fibrosis, and cardiac remodeling.^3 - 5^

The insertion/deletion (I/D) polymorphism of ACE (rs4340), located in the ACE gene (OMIM 106180) on chromosome 17, influences the insertion (allele I) or deletion (allele D) of a 287 base pair alu sequence in intron 16.^3^This polymorphism presents three genotypes (II, DD, and ID), with the I allele associated with reduced enzymatic activity and the D allele with increased activity, correlating with HF exacerbation and the risk of overweight.^3 - 5^Besides influencing BP, ACE inhibits adipocyte differentiation, limiting adipogenesis and adipose tissue storage, resulting in ectopic lipid deposition, affecting cardiac function, and contributing to dysfunction.^4 , 5 - 9^Although previous studies associate ACE polymorphism with predisposition to systemic arterial hypertension (SAH),^10^there is limited literature on its association with HF. This study aims to fill this gap by exploring the interaction between polymorphism, cardiac function, and adiposity in HF patients, to provide insights into disease management, understanding the influence of the D allele and DD genotype on predisposition to overweight and exacerbation of cardiovascular function. The objective of this study is to evaluate adiposity, cardiac function, and their association with ACE polymorphism in HF patients.

## Methods

### Study population

This is a cross-sectional design involving individuals ≥ 18 years of both sexes with an established diagnosis of HF, recruited from November 2022 to October 2023 at a specialized outpatient clinic of a tertiary hospital in southern Brazil. The study consisted of patients with chronic HF of New York Heart Association (NYHA) functional classes I–IV.

Exclusion criteria: a) prior history of acute myocardial infarction; b) liver and kidney failure, undergoing renal replacement therapy; c) type 1 diabetes; and d) inability to perform Bioelectrical Impedance Analysis (BIA), such as amputees or individuals with body mass index (BMI) > 39.9 kg/m^2^.

Socio-demographic data, comorbidities, pharmacological treatment, New York Heart Association (NYHA) functional class, and etiology of HF were extracted from the electronic medical record and confirmed during the research consultation.

This study was approved by the Research Ethics Committee of Hospital de Clínicas de Porto Alegre (CAEE: 63243822.2.0000.5327), following the Declaration of Helsinki. All patients provided written consent.

### Measurement of adiposity and cardiac function

Anthropometric assessment included the use of a digital scale (Toledo®, Araçatuba, São Paulo, Brazil) for body weight and a vertical stadiometer (Veeder-Root® 2.0 m, São Bernardo do Campo, São Paulo, Brazil) to measure height.^11^BMI was categorized according to established cutoff points.^12^

Adiposity was assessed by bioelectrical impedance analysis (BIA) (tetrapolar, model 450 ,800 mA, 50 kHz, Biodynamics Corporation, Seattle, Washington, USA) following standard protocol.^13^The cutoff point for excess adiposity analysis was set at 38% for women and 27% for men relative to fat mass.^14^

Waist circumference (WC) and hip circumference were measured with an inelastic measuring tape. Cardiovascular risk and excess weight were determined based on reference values, including WC > 94 cm for men and 80 cm for women, waist-hip ratio (WHR) ≥ 1.0 for men and > 0.85 for women, and waist-to-height ratio (WHtR) > 0.5 for men and women.^15 - 18^

Two-dimensional echocardiography results using the Simpson method were employed to determine left ventricular ejection fraction (LVEF). This measure categorizes HF into three groups: reduced ejection fraction (HFrEF) when LVEF is < 40%, mildly reduced ejection fraction (HFmrEF) with LVEF between 40-49%, and preserved ejection fraction (HFpEF) when LVEF is ≥ 50%.^1 , 2^

### Genetic analysis

Blood samples (4 mL) with EDTA as an anticoagulant were collected and stored. DNA extraction was performed using the Easy DNA Purification kit™ (Invitrogen™), followed by ACE polymorphism amplification by polymerase chain reaction (PCR). PCR products were genotyped by agarose gel electrophoresis. The primers used were: 5’-CTG GAG AGC CAC TCC CAT CCT TTC T-3’ and 5’-GAC GTG GCC ATC ACA TTC GTC AGA T-3’. PCR conditions were as follows: initial denaturation at 95 °C for 5 minutes; followed by cycles at 95 °C for 40 seconds, 62 °C for 45 seconds, 72 °C for 45 seconds, and a final extension at 72 °C for 5 minutes. ACE polymorphism fragments with deletion (D allele) and without deletion (I allele) of 190 and 490 base pairs, respectively, were visualized on a 1.5% agarose gel containing GelRed nucleic acid stain (Sigma-Aldrich).

### Statistical analysis

Continuous variables were described as mean ± standard deviation or median and interquartile range (25th - 75th percentiles) according to data normality, which was assessed using the Shapiro-Wilk test. Unpaired Student’s t-test or One-way ANOVA with post-hoc Tukey for independent samples was applied to compare means. In case of asymmetry, the Mann-Whitney or Kruskal-Wallis test was used. However, in the Kruskal-Wallis test, Dunn’s post hoc test was not conducted because there was no p-value < 0.05. Categorical variables were described by absolute/relative frequency and Pearson’s Chi-square test or Fisher’s exact test was used to evaluate associations. If there was significance, the residual analysis identified associations. Genetic frequencies were tested for Hardy-Weinberg equilibrium.

The groups were separated according to the three genotypes (II, DD, and ID) of the ACE polymorphism. To analyze the independent effect of the D allele, participants were divided into two groups: those with the presence of the D allele (DD + ID genotypes) and those without the D allele (II genotype only).

The interaction of ACE alleles and variables of interest was analyzed by Poisson regression, with a robust estimator used to control confounding factors. In the multivariate analysis, variables of interest with a p-value < 0.20 in the univariate analysis were considered.

The adopted significance level was 5% (p < 0.05) and analyses were performed using SPSS 21.0 (IBM Corp., Armonk, NY, USA).

## Results

A total of 71 patients were included, with 66.2% being male, with a mean age of 55.8 ± 13 years. The majority of patients presented with NYHA functional classes I and II (90.9%) and a median LVEF of 30% (24-40). Regarding the prevalence of overweight and obesity, 38% of patients were overweight, 23.9% had class I obesity, and 12.7% had class II and III obesity. BIA analysis revealed that 50.7% of patients had excess adiposity. Anthropometric parameters including WC, WHR, and WHtR indicated an increased cardiovascular risk and excess weight, as shown in Table 1 .

**Table 1:** – Clinical, demographic, and anthropometric characteristics of ACE polymorphism genotypes and presence or absence of the D allele in individuals with heart failure

		Genotype				D Allele		
	Total	DD	ID	II	p	Presence	Absence	p
**Number of Patients**	71	27	34	10		61	10	
**Gender (%)**								
Female	24 (33.8)	6 (22.2)	16 (47.1)	2 (20.0)	0.077^§^	22 (36.1)	2 (20.0)	0.477^§^
Male	47 (66.2)	21 (77.8)	18 (52.9)	8 (80.0)		39 (63.9)	8 (80.0)	
**Age (years)**	55.8 ± 13.0	57.2 ± 12.3	56.1 ± 12.4	50.8 ± 16.7	0.404*	56.6 ± 12.3	50.8 ± 16.7	0.191^†^
**Year of Education**	8 (4.0 – 11.0)	8 (4.0 – 11.0)	8 (4.0 – 11.0)	8 (4.0 – 9.0)	0.940^‡^	8 (4.0 – 11.0)	8 (4.0 – 9.0)	0.752^//^
**Ethnicity (%)**								
White	64 (90.1)	24 (88.9)	31 (91.2)	9 (90.0)	1.000^§^	55 (90.2)	9 (90.0)	1.000^§^
Non-White	7 (9.9)	3 (11.1)	3 (8.8)	1 (10.0)		6 (9.8)	1 (10.0)	
**Number of Comorbidities**	2 (1.0 – 3.0)	1 (1.0 – 3.0)	2 (1.0 – 2.0)	0.5 (0.0 – 3.0)	0.321^‡^	2 (1.0 – 2.5)	0.5 (0.0 – 3.0)	0.198^//^
**Comorbidities (%)**								
DM	20 (28.2)	9 (33.3)	8 (23.5)	3 (30.0)	0.693^§^	17 (27.9)	3 (30.0)	1.000^§^
SAH	26 (36.6)	8 (29.6)	14 (41.2)	4 (40.0)	0.631^§^	22 (36.1)	4 (40.0)	1.000^§^
CKD	7 (9.9)	3 (11.1)	3 (8.8)	1 (10.0)	1.000^§^	6 (9.8)	1 (10.0)	1.000^§^
DLP	5 (7.0)	3 (11.1)	2 (5.9)	0 (0.0)	0.688^§^	5 (8.2)	0 (0.0)	1.000^§^
**Time Since Diagnosis of HF (%)**								
≤ 18 months	16 (24.6)	5 (19.2)	6 (20.0)	5 (55.6)	0.067^§^	11 (19.6)^a^	5 (55.6)b	**0.034** ^**§**^
≥ 18 months	49 (75.4)	21 (80.8)	24 (80.0)	4 (44.4)		45 (80.4)^b^	4 (44.4)a	
**HF Etiology (%)**								
Ischemic	7 (11.1)	5 (20.80)a	1 (3.40)a	1 (10.0)a	**0.009** ^**§**^	6 (11.3)^a^	1 (10.0)^a^	**0.025** ^**§**^
Dilated Cardiomyopathy	12 (19.0)	1 (4.20)^a^	6 (20.70)^b^	5 (50.0)^a.b^		7 (13.2)^a^	5 (50.0)^b^	
Other Etiologies	44 (69.8)	18 (75.50)a	22 (75.90)a	4 (40.0)a		40 (75.5)^a^	4 (40.0)^b^	
**LVEF (%)**	30 (24.0 – 40.0)	32 (25.0 – 45.0)	30 (23.0 – 40.0)	24 (18.5 – 33.2)	0.114^‡^	31 (24.5 – 41.0)	24 (18.5 – 33.2)	**0.050** ^**//**^
**LVEF Classification (%)**								
HRrEF	51 (71.8)	17 (63.0)	25 (73.5)	9 (90.0)	0.682^§^	42 (68.9)	9 (90.0)	0.564^§^
HFmrEF	12 (16.9)	6 (22.2)	5 (14.7)	1 (10.0)		11 (18.0)	1 (10.0)	
HFpEF	8 (11.3)	4 (14.8)	4 (11.8)	0 (0.0)		8 (13.1)	0 (0.0)	
**NYHA Classification (%)**								
I and II	60 (90.9)	25 (96.2)	30 (90.9)	5 (71.4)	0.137^§^	55 (93.2)	5 (71.4)	0.118^§^
III and IV	6 (9.1)	1 (3.8)	3 (9.1)	2 (28.6)		4 (6.80)	2 (28.6)	
**Number of Medications**	7 (6.0 – 9.0)	8 (6.0 – 11.0)	7 (5.7 – 9.2)	6.5 (5.7 – 8.0)	0.365^‡^	7 (6.0 –10.0)	7 (4.0 – 8.3)	0.294^//^
**Medications (%)**								
ACEi/ARB/ARNI	58 (81.7)	24 (88.9)	26 (76.5)	8 (80.0)	0.443^§^	50 (82.0)	8 (80.0)	0.881^§^
Beta-Blockers	69 (97.2)	26 (96.3)	33 (97.1)	10 (100.0)	1.000^§^	59 (96.7)	10 (100.0)	1.000^§^
Diuretics	58 (81.7)	23 (85.2)	58 (81.7)	7 (70.0)	0.584^§^	51 (83.6)	7 (70.0)	0.376^§^
Spironolactone	56 (78.9)	22 (81.5)	25 (73.5)	9 (90.0)	0.448^§^	48 (77.0)	9 (90.0)	0.448^§^
**eGFR (ml/min)**	71.0 ± 26.3	69.8 ± 27.2	68.3 ± 25.8	83.2 ± 25.0	0.286*	69.0 ± 26.2	83.2 ± 25.0	0.115^†^
**Creatinine (mg/dL)**	1.0 (0.8 –1.4)	1.2 (0.9 – 1.4)	1.1 (0.8 – 1.5)	1.0 (0.7 – 1.2)	0.557*	1.2 (0.9 – 1.5)	1.0 (0.7 –1.3)	0.305^‡^
**Weight (kg)**	79.9 ± 17.1	84.1 ± 15.5	76.2 ± 17.0	81.1 ± 19.7	0.189*	79.7 ± 16.8	81.1 ± 19.7	0.812^†^
**BMI (kg/m^2^)**	28.8 ± 4.9	30.0 ± 4.5	27.9 ± 5.3	28.6 ± 4.9	0.274*	28.9 ± 5.0	28.6 ± 4.9	0.878^†^
**BMI Classification (%)**								
Normal	18 (25.4)	4 (14.8)	12 (35.3)	2 (20.0)	0.169^§^	16 (26.2)	2 (20.0)	0.224^§^
Overweight	27 (38.0)	12 (44.4)	12 (35.3)	3 (30.0)		24 (39.3)	3 (30.0)	
Obesity Class I	17 (23.9)	5 (18.5)	7 (20.6)	5 (50.0)		12 (19.7)	5 (50.0)	
Obesity Class II and III	9 (12.7)	6 (22.2)	3 (8.8)	0 (0.0)		9 (14.8)	0 (0.0)	
**WC (cm)**	101.4 ± 13.3	102.2 ± 13.3	102.2 ± 13.3	102.2 ± 13.3	0.145*	101.33 ± 13.0	102.4 ± 15.6	0.832^†^
**WHR (cm)**	0.9 ± 0.1	1.0 ± 0.1	0.9 ± 0.1	0.9 ± 0.1	0.218*	0.9 ± 0.1	0.9 ± 0.1	0.968^†^
**WHtR (cm)**	0.6 ± 0.1	0.6 ± 0.1	0.6 ± 0.1	0.6 ± 0.1	0.241*	0.6 ± 0.7	0.6 ± 0.7	0.892^†^
**Body Fat (%)**	29.9 ± 6.3	29.7 ± 5.9	30.7 ± 6.9	28.1 ± 5.2	0.528*	30.2 ± 6.5	28.1 ± 5.2	0.327^†^
Men	27.7 ± 5.0	27.7 ± 4.9	27.6 ± 5.7	27.6 ± 3.7	0.997^§^	27.7 ± 5.2	27.6 ± 3.7	0.944^§^
Women	34.81 ± 6.2	36.7 ± 3.1	34.8 ± 6.6	30.3 ± 11.6	0.461^§^	35.2 ± 5.7	30.3 ± 11.6	0.298^§^
**Adiposity Classification (%)**								
Normal	34 (49.3)	14 (51.9)	15 (46.9)	5 (50.0)	0.929^§^	29 (49.2)	5 (50.0)	1.000^§^
Excess Adiposity	35 (50.7)	13 (48.1)	17 (53.1)	5 (50.0)		30 (50.8)	5 (50.0)	
**Body Fat (kg)**	24.5 ± 8.2	25.3 ± 7.8	24.1 ± 8.6	23.2 ± 8.4	0.754*	24.7 ± 8.2	23.2 ± 8.4	0.613^†^

Data expressed as mean ± standard deviation or median and (25th and 75th percentiles). *One-way ANOVA;^†^Student’s t-test;^‡^Kruskal-Wallis;^§^Chi-square Test or Fisher’s Exact Test;^//^Mann-Whitney; Presence of D allele: DD + ID genotypes; Absence of D allele: II genotype; Statistically significant association by adjusted residuals test at 5% significance level; different letters = statistically significant difference between groups p < 0.005; ARNI: angiotensin receptor–neprilysin inhibitor; DM: Diabetes Mellitus; SAH: systemic arterial hypertension; CKD: chronic kidney disease; DLP: dyslipidemia; ACEI: angiotensin-converting enzyme inhibitor; ARB: angiotensin receptor blocker; LVEF: left ventricular ejection fraction; HFrEF: heart failure with reduced ejection fraction; HFmrEF: heart failure with mid-range ejection fraction; HFpEF: heart failure with preserved ejection fraction; NYHA: new york heart association; eGFR: glomerular filtration rate; WC: waist circumference; WHR: waist-to-hip ratio; WHtR: waist-to-height ratio.

### Genotype and allele frequencies

The allelic frequency was 88 D alleles and 54 I alleles. The genotypic distributions were 38.1% for DD, 47.8% for ID, and 14.1% for II. The frequencies of the ACE I/D polymorphism were in Hardy-Weinberg equilibrium.

### Clinical and anthropometric characteristics in the ace polymorphism

Univariate analysis was conducted to evaluate potential confounding variables ( Table 1 of supplementary material). In the multivariate analysis, when evaluating the association of the D allele with the variables of interest, it was found that LVEF (p = 0.048) and the etiology of dilated cardiomyopathy (DCM) (p = 0.021) were associated with the presence of the D allele (DD+ID versus II) compared to individuals without the D allele. When specifically evaluating the influence of the DD genotype among the other genotypes, it was found that DCM (p = 0.020) and ischemic etiology (p = 0.024) were associated with the DD genotype compared to the ID genotype. Additionally, these associations between LVEF (p = 0.013), DCM etiology (p < 0.001), and the DD genotype persist when compared to the II genotype. Other variables, such as nutritional status, WC, WHR, WHtR, and percentage of body fat, did not present significant associations in multivariate analysis, as described in Table 2 .

**Table 2:** – Multivariate Poisson regression analysis between ACE polymorphism genotypes and the presence or absence of the D allele, clinical characteristics, and adiposity in individuals with heart failure

		DD + ID vs II			DD vs ID			DD vs II	
	**PR**	**CI 95%**	p*	**PR**	**CI 95%**	**p^†^**	**PR**	**CI 95%**	**p^‡^**
**LVEF (%)**	0.995	0.991 - 1.000	**0.048**	0.996	0.990 - 1.001	0.118	0.989	0.980 - 0.998	**0.013**
**LVEF Classification (%)**									
HFrEF	1.145	1.040 - 1.261	**0.006**	1.193	0.941 0 1.512	0.144	1.411	1.132 - 1.758	**0.002**
HFmrEF	1.060	0.925 - 1.216	0.402	1.060	0.782 - 1.436	0.709	1.166	0.926 - 1.468	0.192
HFpEF	1			1			1		
**HF Etiology (%)**									
Ischemic	1.047	0.820 - 1.337	0.714	0.755	0.591 - 0.964	**0.024**	0.969	0.747 - 1.257	0.813
Dilated Cardiomyopathy	1.283	1.039 - 1.583	**0.021**	1.216	1.032 - 1.433	**0.020**	1.584	1.260 - 1.992	**< 0.001**
Other etiologies	1			1			1		
**BMI (kg/m^2^)**	0.999	0.986 - 1.013	0.879	0.988	0.972 - 1.005	0.154	0.968	0.968 - 1.015	0.469
**BMI Classification (%)**									
Normal	1			1			1		
Overweight	0.985	0.832 - 1.165	0.857	0.873	0.727- 1.047	0.143	0.958	0.686 - 1.338	0.802
Obesity Class I	1.169	0.952 - 1.437	0.136	0.893	0.733- 1.087	0.259	1.212	0.868- 1.692	0.260
Obesity Class II and III	0.906	0.789 - 1.039	0.157	0.774	0.573 - 1.046	0.096	0.806	0.605 - 1.073	0.140
**WC (cm)**	1.000	0.994 - 1.006	0.969	0.995	0.988 - 1.002	0.132	0.999	0.989 - 1.008	0.763
**WHR**	0.815	0.269 - 2.473	0.718	0.729	0.216 - 2.456	0.610	0.658	0.137 - 3.168	0.602
**WHtR**	0.882	0.333 - 2.335	0.800	0.432	0.146 - 1.282	0.131	0.572	0.121 - 2.711	0.482
**Body Fat (%)**	0.989	0.973 - 1.005	0.185	0.998	0.983 - 1.014	0.837	0.993	0.970 - 1.017	0.563

Prevalence Ratio (PR) and 95% Confidence Interval (CI); HF: heart failure; LVEF: left ventricular ejection fraction; HFrEF: heart failure with reduced ejection fraction; HFmrEF: heart failure with mid-range ejection fraction; HFpEF: heart failure with preserved ejection fraction; BMI: body mass index; WC: waist circumference; WHR: waist-to-hip ratio; WER: waist-to-height ratio. Multivariable model - LVEF: adjusted for sex and beta-blocker*,^‡^or ACEi/ARB/ARNI^†^; LVEF classification, HF etiology: adjusted for sex and beta-blocker*,^‡^or ACEi/ARB/ ARNI^†^; Body fat, WC, WHR, WER, BMI, Nutritional status: adjusted for sex, beta-blocker*,^‡^or ACEi/ARB/ ARNI^†^, and LVEF classification*,^‡^or HF etiology^†^.

## Discussion

The study’s main findings are the association between the presence of the D allele and the DD genotype of the ACE gene with LVEF and etiology in patients with HF. Adiposity excess was prevalent in the sample, however, no independent associations with polymorphism were identified.

In contrast to previous studies that indicated a reduction in LVEF in patients with the DD genotype,^3 , 18 , 19^our study presents a different perspective, indicating that the median LVEF is lower in patients without the presence of the D allele, possibly related to shorter diagnosis time. This is because D allele carriers have a longer diagnosis time, and as a result, neuro-humoral blockade (pharmacogenetic interaction between beta-blocker and ACE inhibitors use)^19^may have neutralized the excessive activity of the RAAS secondary to DD, influencing this difference between the groups. However, when analyzing the association between the presence of the D allele or the DD genotype versus II, LVEF was observed to increase in association with a decrease in the prevalence of the allele. Therefore, the clinical relevance of this finding implies that patients with the D allele and DD genotype, when compared to II, show a more pronounced deterioration in cardiac function.

The genotypic profile of ACE in the studied population shows a low prevalence of the II genotype compared to international studies, where a prevalence of 23%^20^was identified. When comparing these results with national studies, the prevalence of II was even lower, representing only 3 to 4.5% of the population.^3 , 18^Given that this genomic alteration is relatively recent, the insertion of this Alu sequence is not yet fully established in the human genome, also exhibiting variations in distribution among different ethnic groups.^21^Therefore, this low prevalence of the II genotype may be related to this population characteristic.

Our study revealed a predominance of individuals of European descent (90.1%). In contrast, national studies conducted in other regions of the country^3 , 18^showed a higher percentage of individuals of African descent (14.1% to 36%) and other ethnicities (12.6% to 16.2%). This highlights the epidemiological heterogeneity present in Brazil; however, the southern region has a higher proportion of individuals of European descent. Regarding the other genotypes, there was alignment with European data, showing a higher prevalence of ID genotype.^19^

Another finding was the association of DCM etiology with an increased prevalence of the D allele and DD genotype in HF patients, corroborating previous^22 , 23^findings that established this relationship between the ACE polymorphism and systolic dysfunction of the left ventricle. This can be attributed to more intense neuro-humoral activation of the RAAS.^3 , 18 , 19^The ACE I/D polymorphism plays a significant role, contributing about 50% to the variation in circulating ACE levels.^22 , 23^The D allele intensifies ACE activity, increasing angiotensin synthesis and potentiating RAAS activation, resulting in cardiac dilation.^24^Additionally, the ischemic etiology of HF was associated with a reduction in the prevalence of the DD genotype compared to ID, possibly due to the absence of ACE polymorphism influence in ischemic heart diseases.^25^

Although the DD genotype has been previously associated with overweight/obesity risk in the general population^8^, due to its interaction with adipocyte growth and function, the relationship between polymorphism and overweight was explored in hypertensive patients, presenting conflicting results.^24 , 26^However, in the present study, no independent association of polymorphism with adiposity was observed, which may be due to the high prevalence of overweight/obesity in the sample compared to other populations. The differences in the average BMI of the ACE genotypes between our study and that of Sun et al.^24^were, respectively: 30.0 kg/m^2^vs 26.1 kg/m^2^for DD; 27.9 kg/m^2^vs 26.0 kg/m^2^for ID; and 28.6 kg/m^2^vs 25.9 kg/m^2^for II.

The significant prevalence of adiposity excess among HF patients is a concerning factor, given the considerable health threat that obesity represents. The accumulation of adipose tissue, especially in the visceral region and pericardial fat, results in increased cardiac output and workload.^1 , 2 , 8^Recent studies point to a dose-dependent relationship between BMI increase and HF risk, with obesity considered one of the main causes of HfpEF.^6 , 9^In this scenario, it is crucial to implement effective interventions, including nutritional monitoring, aiming to stimulate weight reduction and mitigate risks related to adiposity.

Possible limitations of this study include the restricted number of participants, especially in the II genotype group, possibly related to the severity of the population predominantly composed of individuals with DD and ID genotypes. The gold standard method for body composition analysis is dual-energy X-ray absorptiometry (DEXA). In its absence, BIA was used. However, BIA has some limitations, such as difficulty in accurately distinguishing between extracellular and intracellular water. This can make body mass estimation susceptible to electrolyte imbalances or variations in hydration status, which are common in patients with HF. In our study, specific protocols were adopted to minimize these limitations, and in the presence of edema, the test was not performed. Although this is the first study to evaluate the ACE polymorphism in Brazilian HF patients and several relevant results were obtained with statistical significance, there is an evident need for future studies with a broader sample, capable of exploring these and other variables potentially related to HF severity and alteration in body composition.

## Conclusion

In summary, this study demonstrated that the presence of the D allele of the ACE polymorphism is associated with LVEF and HF etiology. Despite overweight being prevalent in the sample, no independent associations were found.

*Supplemental Materials

For additional information, please click here.
